# DCB vs. DES in aorto-ostial lesions- clinical outcomes and QCA insights from SPARTAN registry

**DOI:** 10.3389/fcvm.2026.1953073

**Published:** 2026-10-21

**Authors:** Rajkumar Natarajan, Natasha Corballis, Karthik Chandrasekharan, Ioannis Merinopoulos, Tharusha Gunawardena, Upul Wickramarachchi, Allan B. Clark, Vassilios S. Vassiliou, Simon C. Eccleshall, SPARTAN DCB consortium

**Affiliations:** 1 Department of Cardiology, Norfolk and Norwich University Hospital, Norwich, United Kingdom; 2 Norwich Medical School, University of East Anglia, Norwich, United Kingdom

**Keywords:** aorto-ostial lesions, complex coronary artery disease, drug coated balloon (DCB), drug eluting stent (DES), late lumen loss (LLL)

## Abstract

**Background:**

Aorto-ostial *de novo* coronary lesions (AOL) represent a technically challenging subset of coronary artery disease with historically high restenosis rates, even with contemporary drug-eluting stent (DES) platforms. Drug-coated balloons (DCB) offer a stent-less alternative strategy; however, data on their use in AOL are limited. We aimed to compare long-term clinical outcomes of DCB vs. DES in *de novo* AOL.

**Methods:**

We identified all patients undergoing percutaneous coronary intervention (PCI) with either DCB or DES for *de novo* AOL as their first procedure between January 2015 and November 2019. The primary endpoint was target lesion failure (TLF), defined as a composite of target lesion revascularisation (TLR), target lesion myocardial infarction (TLMI), and cardiovascular death. Secondary endpoints included the individual components of TLF, target vessel revascularisation and all-cause mortality. Exploratory quantitative coronary angiography (QCA) analysis was performed on any follow up angiograms obtained that were technically feasible. Clinical outcomes were obtained using national outcome datasets.

**Results:**

A total of 197 patients with aorto-ostial lesions were included (DES *n* = 140; DCB *n* = 57). Over a median follow-up of 3.7 years, TLF occurred in 17.1% of DES-treated lesions and 14.0% of DCB-treated lesions (HR: 0.79, 95% CI: 0.35–1.76; *p* = 0.563). Rates of TLR, TVR, TLMI, cardiovascular mortality, and all-cause mortality were similar between groups. Severe calcification and creatinine independently predicted TLF. Follow-up QCA was available in 59 lesions over varying time intervals and demonstrated lower late lumen loss (LLL) following DCB treatment, compared with DES-treated lesions.

**Conclusion:**

In this real-world cohort with aorto-ostial coronary lesions, a DCB-only strategy was feasible and associated with similar long-term outcomes to DES. Exploratory angiographic analyses demonstrated significant late lumen gain following DCB treatment. These findings are hypothesis-generating and support further prospective evaluation of a DCB approach in aorto-ostial coronary disease.

## 1 Introduction

Historically, percutaneous coronary intervention (PCI) of aorto-ostial lesions (AOL) has been associated with anatomical complexity, substantial procedural challenges and adverse clinical outcomes (1–3). AOL subtends a large myocardial territory and are often associated with diffuse coronary atherosclerosis (4, 5). Continuous exposure to hemodynamic shear forces and non-laminar turbulent flow contributes to their increased fibrosis, nodular calcification and eccentricity (6–8). In addition, complex aorto-ostial muscle bundles and variable take-off anatomy profoundly increase procedural complexity and hinder angiographic visualisation, particularly as guiding catheter engagement and device positioning compete for the same limited ostial space (7–9). Despite advances in contemporary drug eluting stent (DES) technology, including thin-strut platforms with high radial strength, and the development of dedicated ostial stenting techniques, stent failure rates have been reported to be as high as 33% (10–19). This is driven by geographic miss, inadequate lesion coverage, underexpansion, malapposition, stent fracture, crushed or floating struts extending into the aorta, unintended stent deformation (USD) and longitudinal stent deformation (LSD) (14, 15, 18, 20, 21). Collectively, these factors contribute to worse late outcomes compared with non-ostial lesions.

In this context, drug coated balloons (DCB) may address the limitations with ostial stenting in selected lesions by reducing issues with geographic miss, protrusion into the aorta, deformation, and future coronary access. Procedurally, a DCB approach may also simplify PCI in anatomically challenging lesions (22–24). However, evidence regarding the long-term safety and efficacy of DCB in AOL is lacking.

Therefore, we sought to evaluate the long-term clinical and angiographic outcomes, including quantitative coronary angiography (QCA) analysis derived lumen changes, following DCB compared to DES in patients undergoing PCI for AOLs.

## 2 Methods

### 2.1 Registry and ethics

The Safety of Paclitaxel-Drug-Coated Balloon Angioplasty of aorto-ostial coronary lesions (SPARTAN-AOL) study is an investigator-initiated, single-centre, retrospective cohort study. At the time of data collection, our centre performed the highest volume of DCB-only PCI in the UK. In our institution, our PCI data are prospectively collected in a dedicated clinical database (Registration: https://clinicaltrials.gov/ct2/show/NCT04482972 Unique identifier: NCT04482972). The study was approved by the Northwest Haydock (17/NW/0278), Research Ethics Committee (REC), and the Institutional Review Board of the Norfolk and Norwich University Hospital. The Confidentiality Advisory Group (CAG) waived the requirement for patient consent due to the retrospective nature of our study (17/CAG/0145). With the approval of the REC, CAG and local approval, we interrogated the database using two national data storage warehouses from NHS Digital, Hospital Episode Statistics (HES), and National Institute of Cardiovascular Outcome Research (NICOR). This provided the International Classification of Diseases (ICD)-10 codes, which were used to identify patient outcomes, ensuring that all hospital admissions, including those outside of our centre were reported.

### 2.2 Study design and procedural details

From January 2015 to November 2019, patients who underwent PCI with either a DCB or DES for *de novo* AOL were identified. Patients who presented with cardiac arrest, those requiring intubation and mechanical ventilation, or those in cardiogenic shock were excluded, as their outcomes are determined mainly by the severity of the clinical presentation rather than the treatment strategy. Cases requiring both DCB and DES treatment within the same target lesion were excluded to ensure a distinct comparison between treatment strategies. Patients who opted out of HES data sharing for research were excluded.

Aorto-ostial lesions were defined as *de novo* coronary stenoses involving the ostium of the right coronary artery (RCA) or left main stem (LMS), located within 5 mm of the vessel origin from the aortic wall, as assessed angiographically (3). Lesions were further categorised as “true ostial” when the stenosis involved the coronary ostium or within 5 mm from the orifice and the remainder were “treated back to ostium”, defined as lesions originating more distally but received treatment (either DES or DCB) extending to the coronary ostium. Calcification severity was assessed angiographically and categorised as mild, moderate or severe according to standard definitions (25). Vessel diameter was based on the nominal diameter of the largest balloon or stent used. Length of the treated segment was defined as the cumulative length of DCB or DES used to cover the target lesion. Procedural techniques, including lesion preparation and device selection, were left to the operator's discretion in accordance with standard PCI practice. DCB angioplasty was performed according to our previously published algorithm (26).

As no established definitions exist for several of the angiographic characteristics, study-specific definitions were developed by the investigators for the purposes of this study. Hinge motion was defined as visible systolic excursion of the coronary ostium relative to the aortic wall (3). Landing projections were classified according to the angiographic acquisition angle used for device positioning (27). Rotation was categorised as mild (0–19°), moderate (20–39°), or extreme (≥40°). Cranial/caudal tilt was categorised as moderate (15–24°) or extreme (≥25°). Coaxial quality was assessed according to guide catheter stability during contrast injection, ability to identify the ostial landing plane, and susceptibility to guide disengagement during device delivery, and categorised as good, moderate, or poor (28). Device landing was assessed angiographically and classified according to the relationship between the device edge and the ostial plane. Device landing was grouped as landing at the ostial plane or with ≤2 mm aortic protrusion, or as under-landing (≥1 mm within the coronary vessel from the true ostium) or excessive aortic protrusion (≥3 mm) (3, 27, 28). An ostial shelf was defined as a visible step-up between the aortic wall and coronary ostium.

### 2.3 QCA analysis

Routine angiographic follow-up was not mandated within the registry. Follow-up coronary angiography was performed according to clinical indication, including recurrent symptoms, suspected ischaemia, acute coronary syndrome, staged revascularisation, or other clinician-directed reasons. In patients undergoing repeat angiography, follow-up angiograms were assessed for serial quantitative coronary angiography (QCA) analysis.

Retrospective two-dimensional QCA (2D-QCA) was performed using validated CAAS Workstation 8.5 offline analysis software (Pie Medical Imaging BV, Maastricht, Netherlands) by two investigators (RN and NC). Inter-observer reproducibility across all QCA measurements was assessed using intraclass correlations (ICCs), which ranged from 0.90 to 0.94.

Quantitative measurements included reference vessel diameter (RVD), minimal luminal diameter (MLD), percentage diameter stenosis (%DS), acute lumen gain (the difference between post- and pre-procedural MLD), late luminal loss (LLL- the difference between post-procedure and follow-up MLD), net lumen gain at follow up (the difference between follow up and baseline pre-procedural MLD). Binary restenosis was defined as a stenosis of at least 50% of the minimal luminal diameter in the target lesion at follow-up. In most cases, reference vessel diameter was obtained only from a point distal to the lesion. Angiographic patterns of restenosis and the underlying mechanisms were also determined. As operator-estimated vessel sizing may differ from QCA- derived measurements (29, 30), an exploratory device-to-vessel sizing was assessed using the ratio of nominal device diameter (DCB or DES) to QCA-derived RVD and we categorised as undersized (<0.90), appropriately sized (0.90–1.10), or oversized (>1.10). Angiographic restenosis patterns were classified according to Mehran classification (31).

### 2.4 Study endpoints

The primary endpoint was target lesion failure (TLF), a composite endpoint of target lesion revascularisation (TLR), target lesion myocardial infarction (TLMI) and cardiovascular death. The secondary endpoints were individual components of composite endpoint, target vessel revascularisation and all-cause mortality. Endpoints were defined according to the Academic Research Consortium (ARC) criteria, with MI attribution determined at the lesion level (TLMI) (32). All outcomes obtained through HES, NICOR and NHS digital were independently adjudicated by two independent reviewers (RN and NC); adjudication of mortality was performed with reviewers blinded to the intervention.

### 2.5 Statistical analysis

The descriptive statistics of baseline factors of the two treatment groups were compared using a two-sample t-test for continuous variables and a Chi-squared test for binary or categorical variables. For the primary and secondary endpoints, the follow-up time was truncated at the time of mortality due to any cause. Kaplan–Meier failure curves were used to present the survival times and Cox proportional hazards models were used to estimate the hazard ratios of each endpoint alongside the relevant confidence interval and *p*-value. Multivariable Cox regression models were adjusted for clinically related variables according to outcome. Model assumptions were checked using Schoenfeld residuals. Sensitivity analyses restricted analysis to individuals with true ostial lesions overall and restricted to RCA and LMS. In an exploratory analysis, we compared the QCA measurements and outcomes between the two treatment groups using two-sample t-test for continuous variables and a Chi-squared test for binary or categorical variables. We also explored the association between late lumen loss and time since the procedure using a scatter graph and linear regression. Significance tests and confidence intervals were two-sided and were set at the 5% and 95% levels respectively.

## 3 Results

### 3.1 Study population and baseline characteristics

A total of 197 patients were included in this study. Of them, 140 were treated with DES and 57 with paclitaxel coated balloon. As summarised in Table 1, both groups were well balanced in their baseline characteristics and co-morbidities. Clinical presentation was similar between groups, with no statistically significant differences in rates of stable angina, STEMI or NSTEMI/unstable angina (all *p* > 0.05).

**Table 1:** Baseline clinical characteristics.

Characteristics	DES (*n* = 140)	DCB (*n* = 57)	*p* value
Age, years (mean ± SD)	69.19 ± 11.08	71.3 ± 12.65	0.25
Male, *n* (%)	91 (65)	44 (77.2)	0.09
Hypertension, *n* (%)	66 (47.1)	30 (52.6)	0.48
Hypercholesterolemia, *n* (%)	33 (23.6)	16 (28.1)	0.51
Diabetes mellitus, *n* (%)	26 (18.6)	15 (26.3)	0.22
Smoking history, *n* (%)	81 (57.9)	33 (57.9)	0.93
Previous MI, *n* (%)	26 (18.6)	9 (15.8)	0.64
Previous PCI, *n* (%)	19 (13.6)	4 (7.0)	0.19
Previous CABG, *n* (%)	20 (14.3)	13 (22.8)	0.15
Peripheral vascular disease, *n* (%)	6 (4.3)	3 (5.3)	0.77
Cerebrovascular disease, *n* (%)	10 (7.1)	6 (10.5)	0.43
COPD, *n* (%)	11 (7.9)	3 (5.3)	0.52
Asthma, *n* (%)	8 (5.7)	6 (10.5)	0.23
Family history of CAD, *n* (%)	17 (12.1)	6 (10.5)	0.75
Stable angina presentation, *n* (%)	55 (39.3)	21 (36.8)	0.75
ACS (STEMI), *n* (%)	29 (20.7)	10 (17.5)	0.61
ACS (NSTEMI or UA), *n* (%)	56 (40.0)	26 (45.6)	0.47
Creatinine (µmol/L, mean ± SD)	93.09 ± 57.27	87.51 ± 22.34	0.48

ACS, acute coronary syndrome; CABG, coronary artery bypass graft; CAD, coronary artery disease; DCB, drug coated balloons; COPD, chronic obstructive pulmonary disease; DES, drug eluting stent; MI, myocardial infarction; NSTEMI, non-ST elevation myocardial infarction; PCI, percutaneous coronary intervention; STEMI, ST elevation myocardial infarction; UA, unstable angina. Values are expressed as number (%), mean ± standard deviation (SD), as appropriate. Statistically significance at *p* < 0.05.

### 3.2 Procedural characteristics

Table 2 outlines procedural and angiographic details. True ostial disease was seen in 156 patients, of which 47 received DCB treatment and 109 were treated with DES. DCB-treated lesions were characterised by greater lesion complexity, including higher degree of angiographic calcification and vessel tortuosity and were more frequently managed with speciality balloons for lesion preparation strategies. Procedural dissections were more commonly seen in DCB strategy. Contrast use and mean vessel/device diameter were significantly higher in DES cohort. DES-treated lesions more often required multiple angiographic views for ostial landing. While protruding ostial landing was broadly similar between groups, greater protrusion length was observed in the DCB group. The DES platforms comprised PROMUS Element, PROMUS Premier, SYNERGY, XIENCE Prime, XIENCE Pro, Resolute Onyx and Ultimaster, while the DCB platforms comprised SeQuent Please and SeQuent Please NEO.

**Table 2:** Angiographic and procedural characteristics.

Characteristics	DES (*N* = 140) *n* (%)	DCB (*N* = 57) *n* (%)	*p*-value
True ostial disease	109 (77.9%)	47 (82.5%)	0.47
Vessel treated			0.27
LMS	69 (49.3%)	33 (57.9%)	
RCA	71 (50.7%)	24 (42.1%)	
Calcification	56 (40.0%)	32 (56.1%)	**0** **.** **039**
Mild	9 (6.4%)	4 (7.0%)	0.20
Moderate	17 (12.1%)	11 (19.3%)	
Severe	30 (21.4%)	17 (29.8%)	
Diffuse disease	31 (22.1%)	8 (14.0%)	0.20
Tortuosity	14 (10.0%)	12 (21.1%)	**0** **.** **038**
Vessel Diameter, mean (SD)	3.95 (0.61)	3.64 (0.43)	**<0** **.** **001**
Small vessel (≤2.75 mm),	0	3 (5.3%)	0.006
Large vessel (≥3.00 mm)	140 (100.0%)	54 (94.7%)	0.006
Multivessel PCI	28 (20.0%)	15 (26.3%)	0.33
Multilesion PCI	26 (18.6%)	9 (15.8%)	0.64
Contrast use, median (IQR)	150.00 (110.00, 190.00)	120.00 (90.00, 160.00)	**0** **.** **002**
Hinge motion present (visible systolic excursion at ostium)	7 (5.0%)	6 (10.5%)	0.16
Landing views			0.54
LAO CRA	37 (26.4%)	14 (24.6%)	
LAO CAU	54 (38.6%)	17 (29.8%)	
RAO CRA	17 (12.1%)	7 (12.3%)	
RAO CAU	14 (10.0%)	7 (12.3%)	
PA CRA	6 (4.3%)	2 (3.5%)	
PA CAU	12 (8.6%)	10 (17.5%)	
Rotation			0.65
Moderate	86 (61.4%)	37 (64.9%)	
Extreme	54 (38.6%)	20 (35.1%)	
No. of views used for landing			**0** **.** **011**
1	101 (72.1%)	52 (91.2%)	
2	33 (23.6%)	5 (8.8%)	
3	6 (4.3%)	0	
Coaxial quality			0.55
Good	121 (86.4%)	46 (80.7%)	
Moderate	10 (7.1%)	5 (8.8%)	
Poor	9 (6.4%)	6 (10.5%)	
Catheter induced trauma	4 (2.9%)	2 (3.5%)	0.81
Device landing			0.37
On plane or ≤2 mm protrusion	100 (71.4%)	37 (64.9%)	
Underlanded or ≥3 mm protrusion	40 (28.6%)	20 (35.1%)	
Ostial shelf (a step up from aortic wall)	17 (12.1%)	14 (24.6%)	**0** **.** **030**
Predilatation balloon max size, mean (SD)	3.27 (0.61)	3.58 (0.46)	**<0** **.** **001**
Direct stenting	15 (10.7%)	0	**0** **.** **010**
Non-compliant balloon	78 (55.7%)	44 (77.2%)	**0** **.** **005**
Scoring balloon	35 (25.0%)	34 (59.6%)	**<0** **.** **001**
Cutting balloon	3 (2.1%)	2 (3.5%)	0.58
Final device diameter following post dilatation, mean (SD)	4.14 (0.64)	3.61 (0.43)	**<0** **.** **001**
Treated lesion length, mean (SD)	22.35 (10.93)	21.74 (9.49)	0.71
Dissection during lesion preparation	2 (1.4%)	23 (40.4%)	**<0** **.** **001**
Protrusion length mm, mean (SD)	0.57 (1.30)	1.16 (2.16)	**0** **.** **021**

CAU, caudal; CRA, cranial; DCB, drug-coated balloon; DES, drug-eluting stent; LAO, left anterior oblique; LMS, left main stem; PA, posteroanterior; PCI, percutaneous coronary intervention; RAO, right anterior oblique; RCA, right coronary artery; SD, standard deviation.

Statistical significant values are expressed in bold.

### 3.3 Clinical outcomes

The median clinical follow-up duration was 3.7 years (IQR: 2.3–4.9) overall, 3.9 years (IQR: 2.2–4.9) for DES group, and 3.7 years (IQR: 2.4–4.9, *p* = 0.975) for DCB group. Clinical outcomes during follow-up, in an unadjusted analysis, are summarised in Table 3. Between the treatment strategies, no significant differences were observed in TLF, TLR, TLMI, TVR, cardiovascular death, or all-cause mortality.

**Table 3:** Estimated unadjusted HR for each outcome estimated from a cox model.

Outcome	DES (*N* = 140) *n* (%)	DCB (*N* = 57) *n* (%)	Unadjusted HR DCB vs. DES (95% CI)	*p*-value
TLF	24 (17.1%)	8 (14.0%)	0.79 (0.35, 1.76)	0.563
TLR	14 (10.0%)	4 (7.0%)	0.68 (0.22, 2.08)	0.502
TLMI	8 (5.7%)	1 (1.8%)	0.30 (0.04, 2.38)	0.254
TVR	14 (10.0%)	4 (7.0%)	0.68 (0.22, 2.08)	0.502
Cardiovascular death	8 (5.7%)	4 (7.0%)	1.23 (0.37, 4.10)	0.733
All-cause mortality	25 (17.9%)	11 (19.3%)	1.07 (0.53, 2.19)	0.844

CI, confidence interval; DCB, drug coated balloon; DES, drug eluting stents; HR, Hazard ratio; N, total number of patients; n, number of events; TLF, target lesion failure; TLR, target lesion revascularisation; TLMI, target lesion myocardial infarction; TVR, target vessel revascularisation.

Kaplan–Meier failure estimates at 5 years demonstrated no significant differences in clinical endpoints between DCB and DES group. Figures 1–6 represents KM failure plots for TLF, TLR, TLMI, TVR, cardiovascular death, and all-cause death respectively.

**Figure 1:**
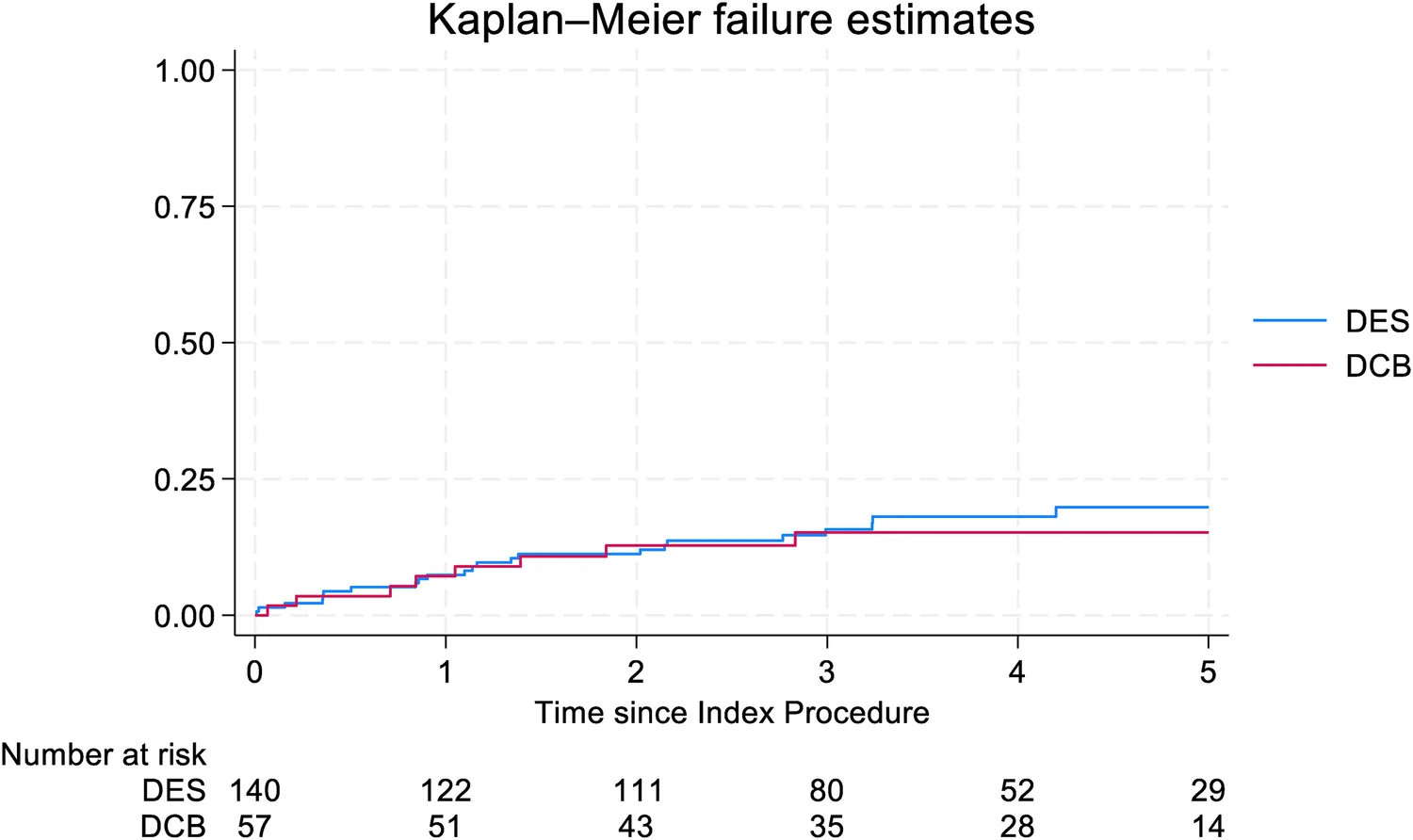
Kaplan–meier failure plot for target lesion failure (TLF). DES, drug-eluting stent; DCB, drug-coated balloon.

**Figure 2:**
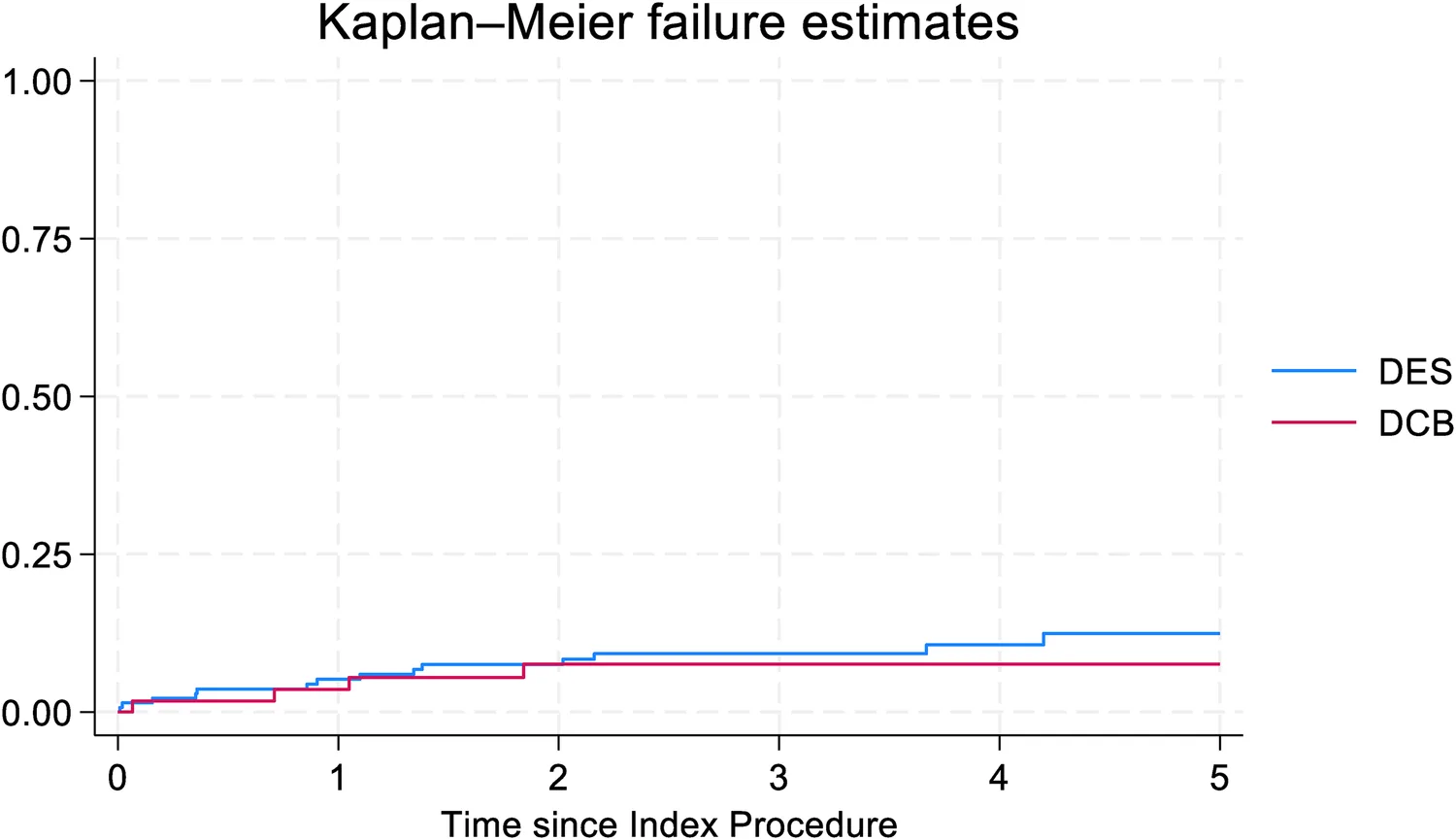
Kaplan–meier failure plot for target lesion revascularisation (TLR). DES, drug-eluting stent; DCB, drug-coated balloon.

**Figure 3:**
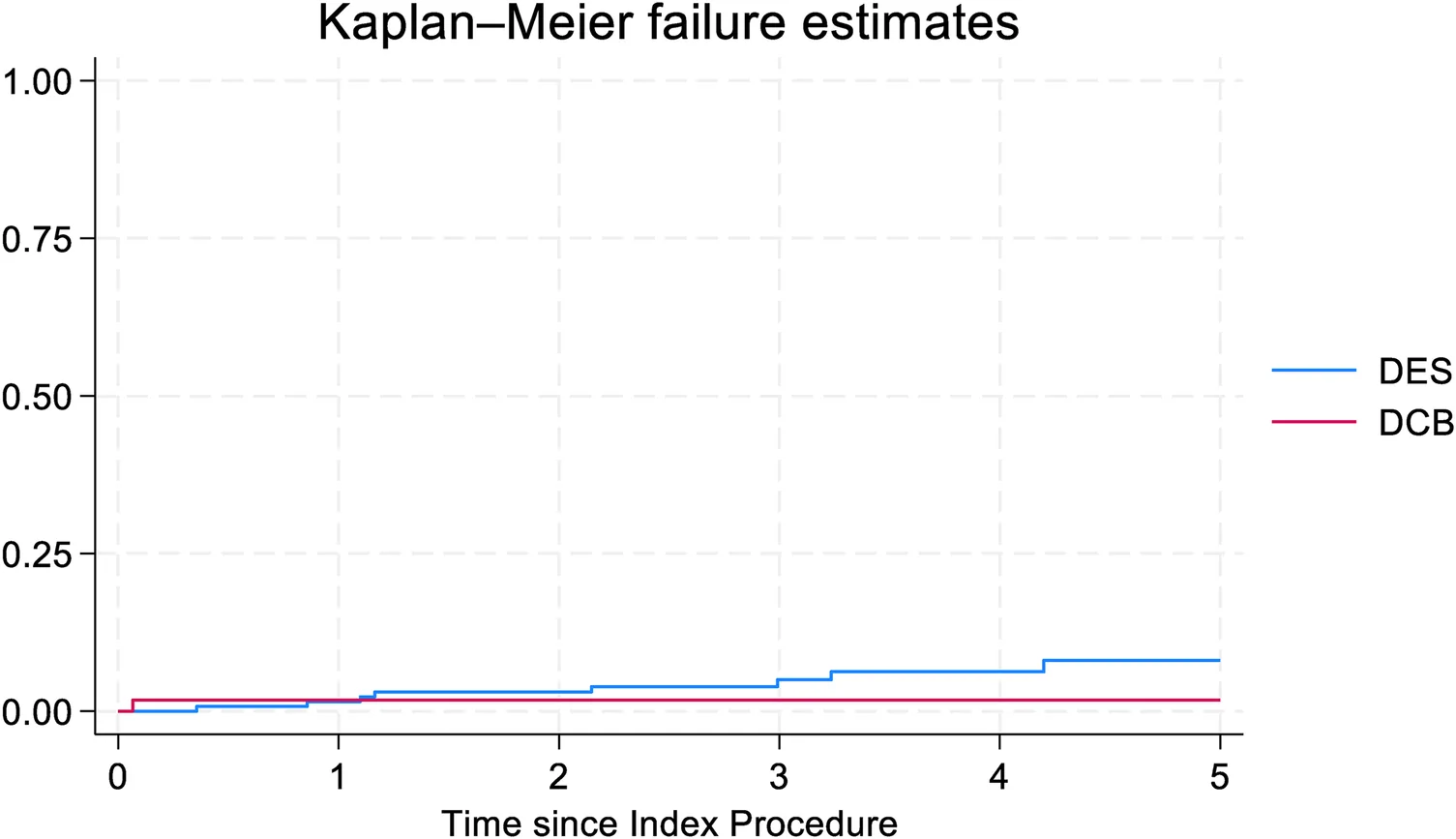
KM failure plot for target lesion-myocardial infarction (TLMI). DES, drug-eluting stent; DCB, drug-coated balloon.

**Figure 4:**
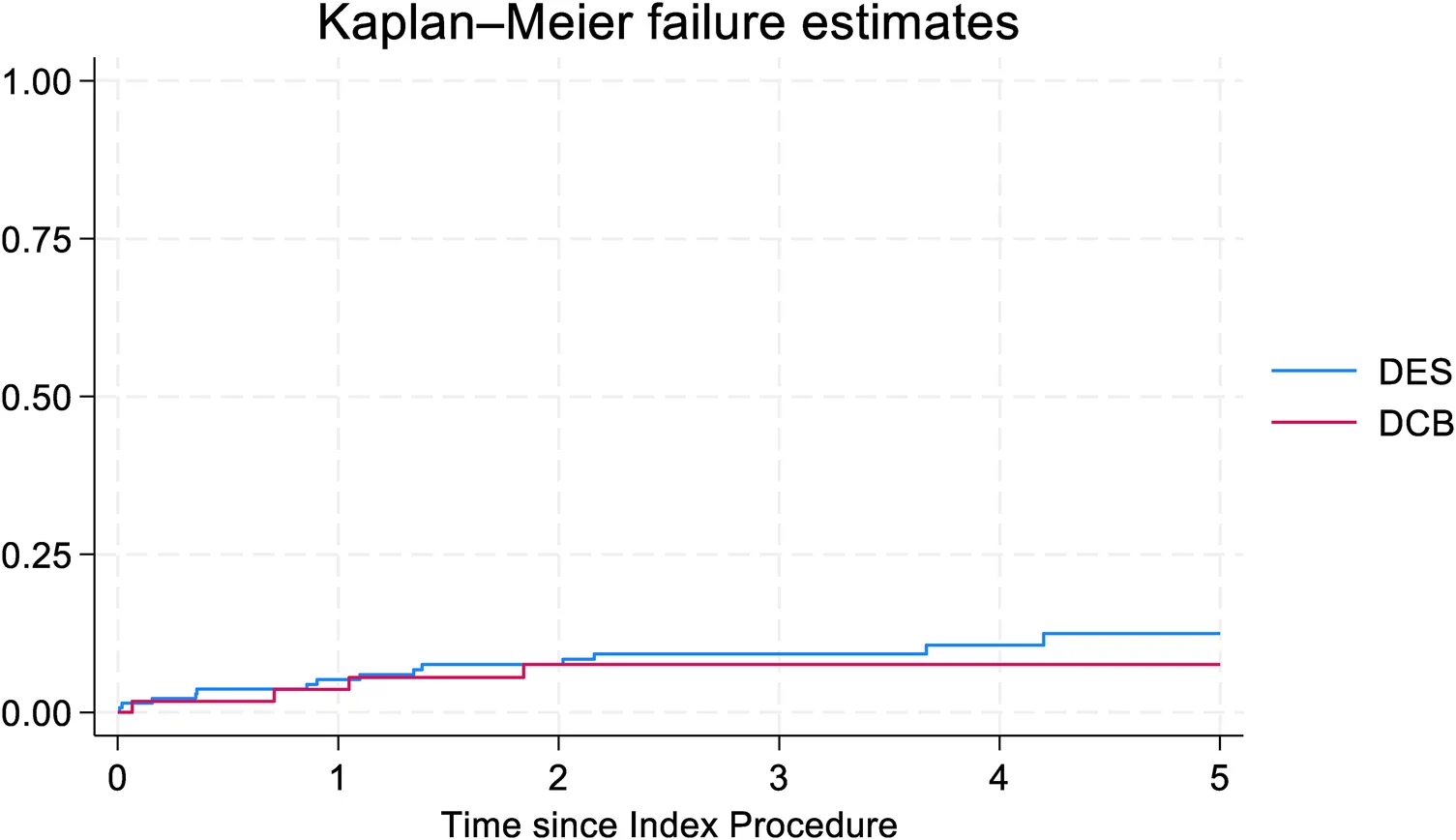
KM failure plot for target vessel revascularisation (TVR). DES, drug-eluting stent; DCB, drug-coated balloon.

**Figure 5:**
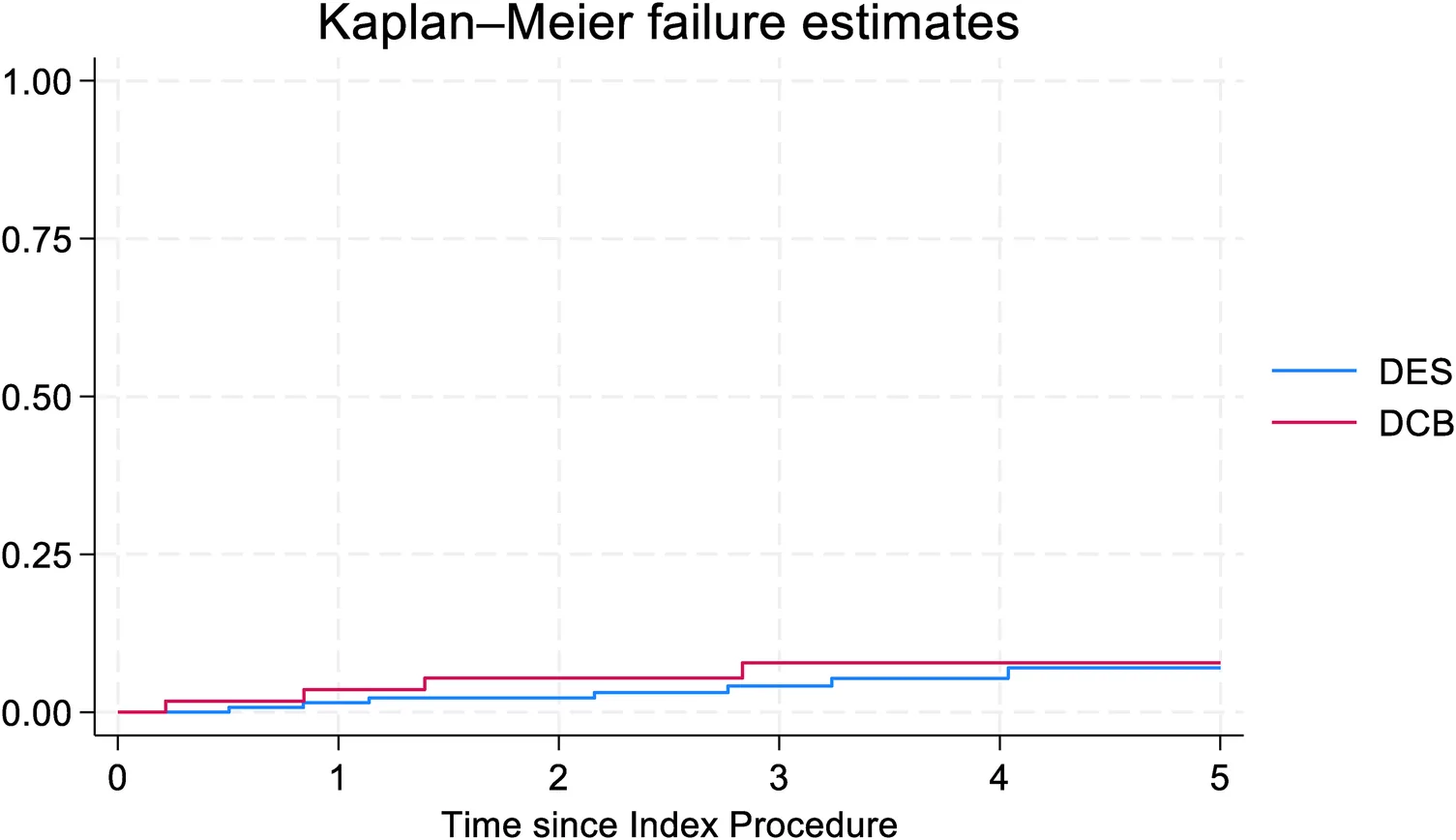
KM failure plot for cardiovascular death. DES, drug-eluting stent; DCB, drug-coated balloon.

**Figure 6:**
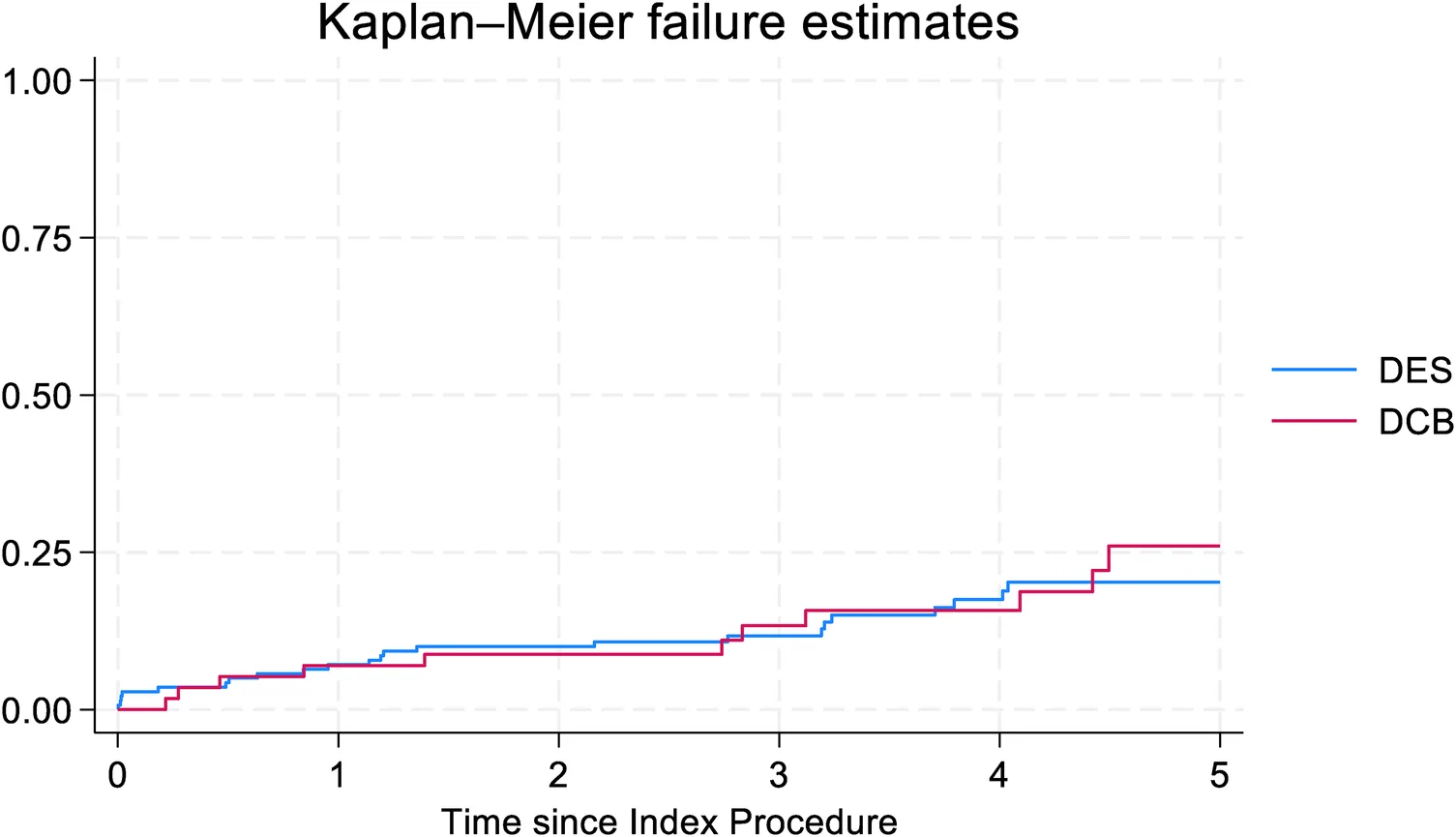
KM failure plot for all-cause mortality. DES, drug-eluting stent; DCB, drug-coated balloon.

#### 3.3.1 Adjusted analyses

Considering the limited number of clinical events, exploratory adjusted cox-regression analyses were performed using a limited number of clinically relevant covariates, as shown in Table 4. For TLF, adjustment for creatinine and calcification demonstrated findings consistent with the primary analysis, with no significant association between treatment strategy and TLF. Severe calcification and increasing creatinine were independently associated with TLF. Similarly, for all-cause mortality, adjustment for age and creatinine did not demonstrate a significant association between treatment strategy and mortality, while increasing age and creatinine were independently associated with mortality.

**Table 4:** Adjusted cox analyses for TLF and all-cause mortality.

Covariates	TLF		All-cause mortality	
	HR (95% CI)	*p*-value	HR (95% CI)	*p*-value
DCB vs. DES	0.79 (0.35 ,1.79)	0.579	0.98 (0.47, 2.02)	0.949
Creatinine (10 unit)	1.04 (1.01 ,1.07)	0.015	1.03 (1.00, 1.06)	0.037
Age	–	–	1.08 (1.03, 1.12)	<0.001
Calcification				
Mild	0.57 (0.07 ,4.33)	0.586	–	
Moderate	0.53 (0.12 ,2.32)	0.396	–	
Severe	2.58 (1.20 ,5.53)	0.015	–	

An exploratory analysis for TLF adjusting for creatinine and calcification results are above. Additionally, for all-cause mortality we adjusted for age and creatinine. CI, confidence interval. DCB, drug coated balloon; DES, drug eluting stents; HR, Hazard ratio; N, total number of patients; n, number of events; TLF, Target lesion failure.

### 3.4 Sensitivity analyses

#### 3.4.1 By true ostial AOL

Estimated unadjusted hazard ratios for the true ostial lesions are presented in Table 5 and demonstrated findings similar to those observed in the whole cohort.

**Table 5:** Estimated HR for the subset of individuals with true aorto-ostial disease.

Outcome	DES (*N* = 109) *n* (%)	DCB (*N* = 47) *n* (%)	HR (95% CI)	*p*-value
TLF	18 (16.5%)	7 (14.9%)	0.85 (0.36, 2.05)	0.724
TLR	10 (9.2%)	3 (6.4%)	0.68 (0.19, 2.45)	0.551
TLMI	5 (4.6%)	0	Not estimable	–
TVR	10 (9.2%)	3 (6.4%)	0.68 (0.19, 2.45)	0.551
Cardiovascular death	6 (5.5%)	4 (8.5%)	1.54 (0.43, 5.46)	0.504
All-cause mortality	20 (18.3%)	8 (17%)	0.92 (0.41, 2.10)	0.846

Sensitivity analyses stratified by true aorto-ostial lesions. For TLMI, HR is not estimable due to zero events in DCB group. CI, confidence interval; DCB, drug coated balloon; DES, drug eluting stents; HR, Hazard ratio; N, total number of patients; n, number of events; NE, non estimable owing to zero events; RCA, right coronary artery; TLF, target lesion failure; TLR, target lesion revascularisation; TLMI, target lesion myocardial infarction.

#### 3.4.2 By vessel treated– LMS vs. RCA

Exploratory sensitivity analyses according to the vessel treated––LMS vs. RCA in the overall cohort, showed no significant differences between groups, as presented in Tables 6, 7.

**Table 6:** Sensitivity analyses restricted to LMS aorto-ostial lesions.

Outcome	DES (*N* = 69) *n* (%)	DCB (*N* = 33) *n* (%)	HR (95% CI)	*p*-value
TLF	10 (14.5%)	7 (21.2%)	1.57 (0.60, 4.13)	0.360
TLR	5 (7.3%)	4 (12.1%)	1.80 (0.48, 6.71)	0.382
TLMI	3 (4.3%)	1 (3.0%)	0.71 (0.07, 6.79)	0.763
TVR	5 (7.3%)	4 (12.1%)	1.80 (0.48, 6.71)	0.382
CVD	4 (5.8%)	3 (9.1%)	1.59 (0.35, 7.11)	0.545
All-cause mortality	14 (20.3%)	8 (24.2%)	1.17 (0.49, 2.80)	0.720

CI, confidence interval; DCB, drug coated balloon; DES, drug eluting stents; HR, Hazard ratio; N, total number of patients; n, number of events; LMS, left main stem; TLF, target lesion failure; TLR, target lesion revascularisation; TLMI, target lesion myocardial infarction.

**Table 7:** Sensitivity analyses restricted to RCA aorto-ostial lesions.

Outcome	DES (*N* = 71) *n* (%)	DCB (*N* = 24) *n* (%)	HR (95% CI)	*p*-value
TLF	14 (19.7%)	1 (4.2%)	0.17 (0.02, 1.32)	0.091
TLR	9 (12.7%)	0	NE	NA
TLMI	5 (7.0%)	0	NE	NA
TVR	9 (12.7%)	0	NE	NA
CVD	4 (5.6%)	1 (4.2%)	0.73 (0.08, 6.55)	0.778
All-cause mortality	11 (15.5%)	3 (12.5%)	0.81 (0.22, 2.91)	0.746

CI, confidence interval; DCB, drug coated balloon; DES, drug eluting stents; HR, Hazard ratio; N, total number of patients; n, number of events; NE, non estimable owing to zero events; RCA, right coronary artery; TLF, target lesion failure; TLR, target lesion revascularisation; TLMI, target lesion myocardial infarction.

Among RCA DES TLRs (*n* = 9), angiographic evidence of stent recoil was identified in one-third of the cases, while the remainder predominantly related to diffuse proliferative in-stent restenosis (Mehran class III), including one episode of late stent thrombosis. In LMS lesions, TLR mechanisms appeared to be slightly different. Among DES-treated LMS lesions (*n* = 5), mechanism of TLR included stent malapposition identified on IVUS (*n* = 1), a calcific nodule at proximal stent stenosis (*n* = 1), diffuse neointimal restenosis (*n* = 1), bifurcation-related failure with ostial circumflex restenosis (*n* = 2). In DCB-treated LMS lesions, focal (Mehran I) or diffuse (Mehran II) in-stent restenosis were seen in TLR cases (*n* = 4).

## 4 QCA analyses

### 4.1 Baseline QCA

Baseline 2D-QCA of index PCI procedure was feasible in 183 patients (DES: *n* = 137; DCB: *n* = 46), limited by the availability and adequacy of angiographic images for reliable analysis.

Table 8 depicts the baseline QCA for the index PCI procedures. Pre-procedural QCA derived parameters were comparable between the groups. Post procedural MLD was lower and residual %DS was significantly higher in DCB cohort.

**Table 8:** Baseline QCA for whole cohort .

Parameter	DES	DCB	*p*-value
No. of patients	137	46	
Group			0.98
True ostial	107 (78.1%)	36 (78.3%)	
Treated back to ostium	30 (21.9%)	10 (21.7%)	
Vessel treated			0.64
LMS	71 (51.8%)	22 (47.8%)	
RCA	66 (48.2%)	24 (52.2%)	
Pre-PCI			
RVD, mm, mean (SD)	3.71 (0.74)	3.66 (0.64)	0.70
MLD, mm, mean (SD)	0.84 (0.66)	0.91 (0.63)	0.55
%DS, mean (SD)	78.09 (15.19)	78.52 (13.56)	0.86
Post PCI			
RVD, mm, mean (SD)	3.95 (0.66)	3.83 (0.67)	0.29
MLD, mm, mean (SD)	3.23 (0.67)	2.76 (0.65)	<0.001
%DS, mean (SD)	17.56 (11.42)	25.67 (11.89)	<0.001

LMS, Left main stem; MLD, minimum lumen diameter; RCA, right coronary artery; PCI, percutaneous coronary intervention; RVD, reference vessel diameter; mm, millimetre; %DS, percent diameter stenosis; SD, standard deviation.

### 4.2 Follow-up QCA exploratory analyses

Serial follow-up 2D-QCA was feasible in 59 patients undergoing repeat angiography (DES: *n* = 42; DCB: *n* = 17), in whom QCA measurements and outcome assessment was performed opportunistically. Overall, follow-up angiography was performed at a median of 0.6 years (IQR: 0.2–3.3) after index PCI. The interval to follow-up angiography was numerically longer in the DES group than the DCB group [1.1 years [IQR: 0.3–3.6] vs. 0.4 year [IQR: 0.2–0.9] years; *p* = 0.08]. Follow-up QCA findings are summarised in Table 9.

**Table 9:** Follow up QCA measurements and outcomes.

Parameter	DES	DCB	*p*-value
No. of patients	42	17	
Time to follow-up angiography, years, median (IQR)	1.1 (0.3–3.6)	0.4 (0.2–0.9)	0.08
Group			0.24
True ostial, *n* (%)	29 (69.0%)	9 (53.0%)	
Treated back to ostium, *n* (%)	13 (31.0%)	8 (47.0%)	
Vessel treated			0.54
LMS, *n* (%)	21 (50.0%)	7 (41.0%)	
RCA, *n* (%)	21 (50.0%)	10 (59.0%)	
Pre-PCI			
RVD, mm, mean (SD)	3.71 (0.97)	3.62 (0.61)	0.70
MLD, mm, mean (SD)	0.87 (0.78)	0.70 (0.50)	0.43
%DS, mean (SD)	77.64 (17.48)	81.88 (11.58)	0.36
Post-PCI			
RVD, mm, mean (SD)	3.91 (0.75)	3.77 (0.65)	0.51
MLD, mm, mean (SD)	3.22 (0.76)	2.66 (0.60)	0.009
%DS, mean (SD)	17.60 (9.77)	30.47 (9.99)	<0.001
Acute lumen gain, mean (SD)	2.36 (0.95)	1.96 (0.67)	0.12
Device to QCA vessel-size ratio, mean (SD)	1.04 (0.17)	0.97 (0.13)	0.14
Follow-up QCA			
RVD, mm, mean (SD)	3.85 (0.76)	3.82 (0.64)	0.87
MLD, mm, mean (SD)	2.42 (1.31)	2.91 (0.81)	0.15
%DS, mean (SD)	39.07 (28.68)	23.41 (19.74)	0.044
Binary restenosis, *n* (%)	12 (29.0%)	2 (12.0%)	0.17
Device:QCA size categories			
Undersized	6 (14.0%)	6 (35.0%)	0.19
Appropriate	21 (50.0%)	6 (35.0%)	
Oversized	15 (36.0%)	5 (29.0%)	
QCA Outcomes			
LLL, mm, mean (SD)	0.81 (1.11)	−0.25 (0.80)	<0.001
Net gain, mm, mean (SD)	1.55 (1.38)	2.21 (0.95)	0.076

Device-to-vessel sizing was assessed using the ratio of nominal device diameter (DCB or DES) to QCA-derived RVD and categorised as undersized (<0.90), appropriately sized (0.90–1.10), or oversized (>1.10). LMS, Left main stem; RCA, right coronary artery; RVD, reference vessel diameter; mm, millimetre; MLD, minimum lumen diameter; %DS, percent diameter stenosis; LLL, late lumen loss (MLD post PCI—MLD follow up); Net gain, MLD follow up—MLD pre-PCI; SD, standard deviation; mm-millimetre; PCI- percutaneous coronary intervention.

At follow-up, DCB-treated lesions had significantly lower %DS than DES-treated lesions (23.41 ± 19.74% vs. 39.07 ± 28.68%; *p* = 0.044). Follow-up MLD was numerically higher after DCB treatment, although this difference was not statistically significant (2.91 ± 0.81 mm vs. 2.42 ± 1.31 mm; *p* = 0.15). Binary restenosis rates were numerically lower in DCB group (12% vs. 29%, *p* = 0.17). Negative LLL (−0.25 ± 0.80) was observed following DCB treatment, whereas DES-treated lesions demonstrated positive LLL (0.81 ± 1.11, *p* < 0.001). Device-to-QCA vessel sizing ratios and sizing categories were broadly comparable between treatment groups.

Exploratory scatter plot analyses assessed the temporal patterns of LLL between treatment strategies. In Figure 7, fitted trend lines suggested a tendency towards late lumen gain following DCB treatment, whereas DES treatment showed progressive late lumen loss over time. When stratified to vessel treated, similar LLL behaviour was seen in both LMS and RCA lesions, while a greater visual separation in LLL trend lines was apparent in RCA lesions (Figures 8, 9). It should be interpreted in the context of differing follow-up durations, with longer angiographic follow-up in LMS compared with RCA lesions.

**Figure 7:**
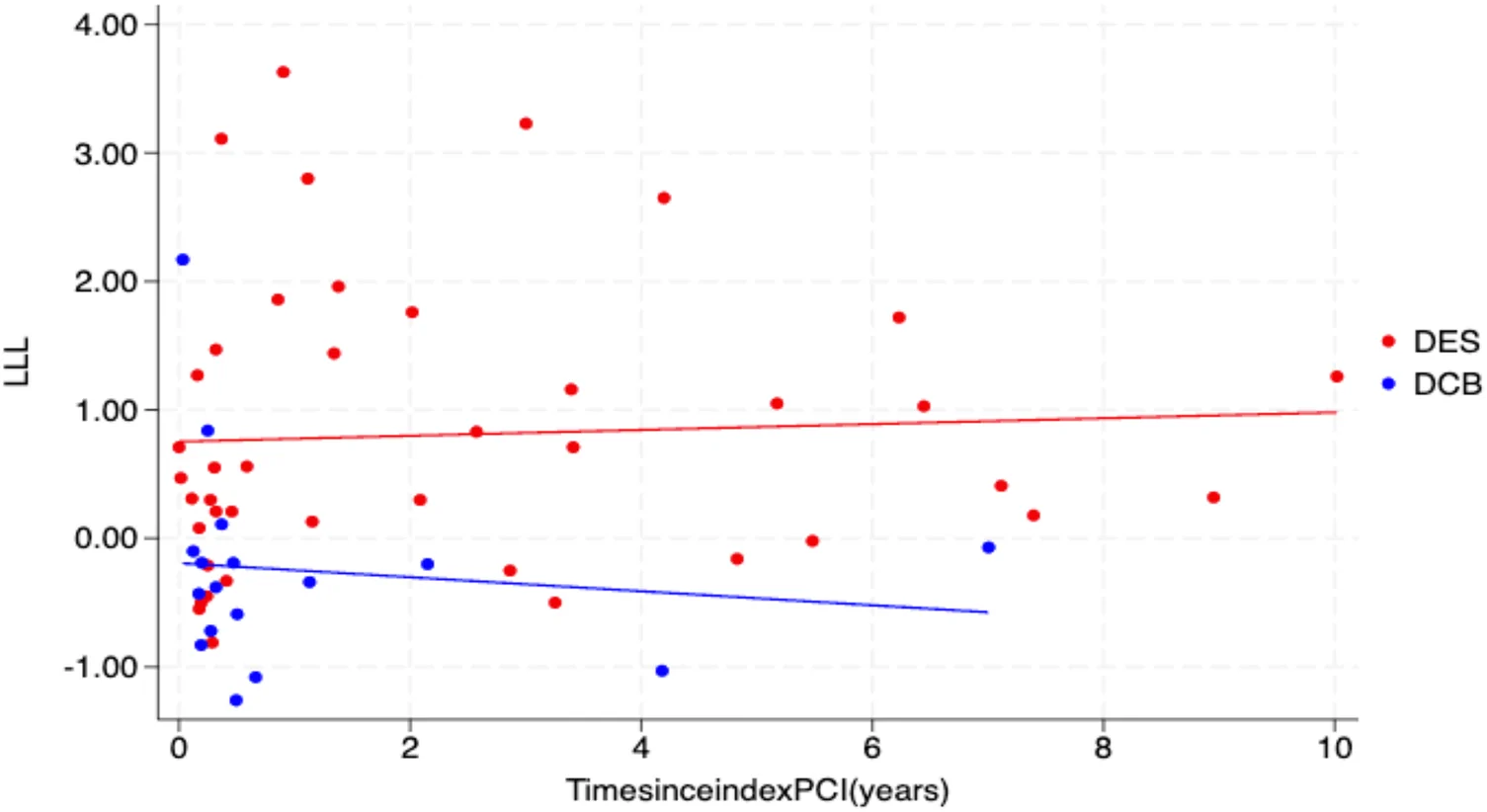
Scatter plot demonstrating late lumen loss (LLL) over time following index PCI according to treatment strategy. Fitted trend lines illustrate temporal patterns of LLL in DES-treated (red) and DCB-treated (blue) lesions. DES, drug eluting stents; DCB, drug coated balloon.

**Figure 8:**
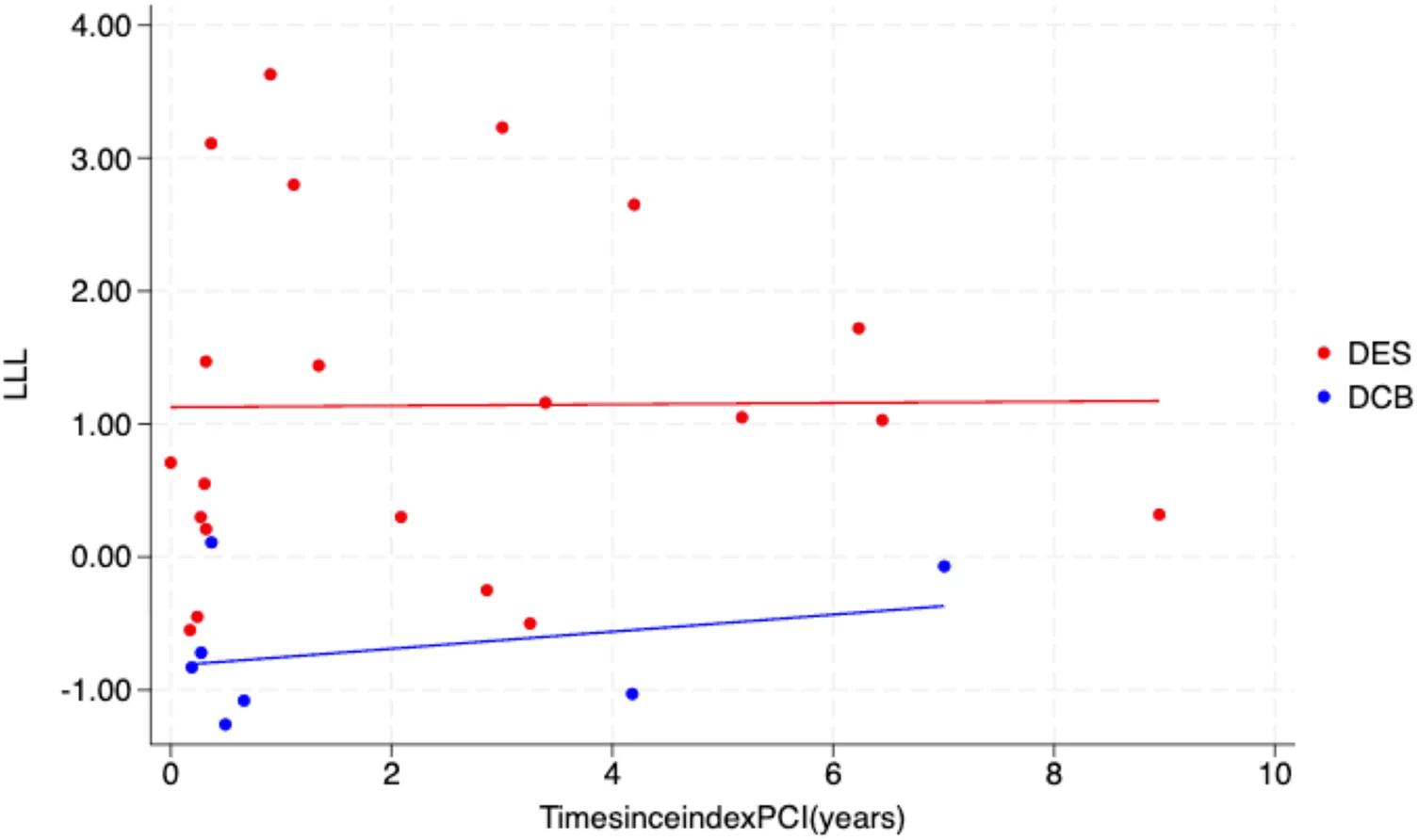
Scatter plot demonstrating late lumen loss (LLL) over time in left main stem (LMS) aorto-ostial lesions according to treatment strategy. Fitted trend lines illustrate temporal LLL patterns in DES-treated (red) and DCB-treated (blue) lesions. DES, drug eluting stents; DCB, drug coated balloon.

**Figure 9:**
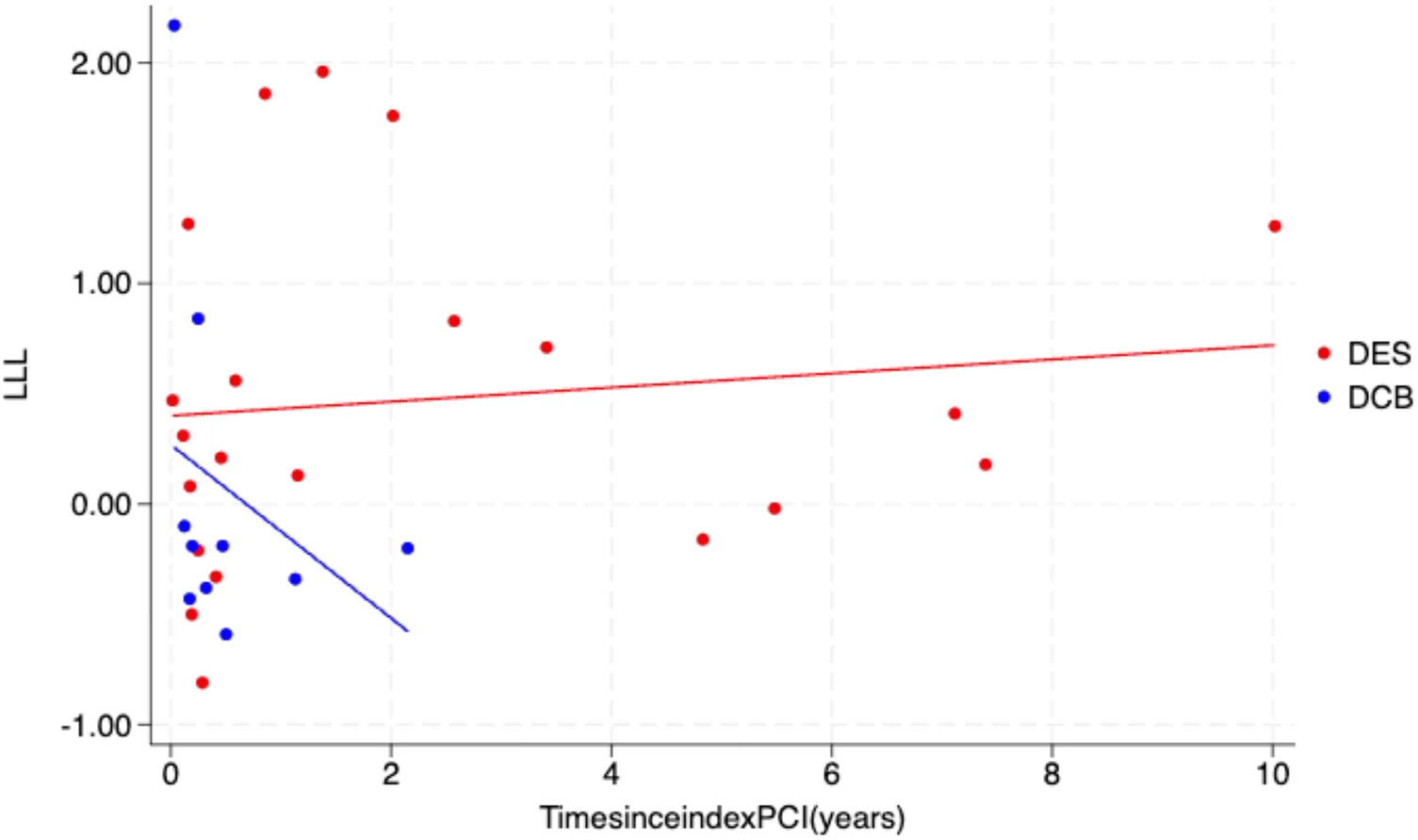
Scatter plot demonstrating late lumen loss (LLL) over time in right coronary artery (RCA) aorto-ostial lesions according to treatment strategy. Fitted trend lines illustrate temporal LLL patterns in DES-treated (red) and DCB-treated (blue) lesions. DES, drug eluting stents; DCB, drug coated balloon.

## 5 Discussion

The principal findings of our study are as follows. First, in this real-world cohort of patients undergoing PCI for AOL, a DCB-only strategy appears safe and feasible with comparable long-term outcomes to DES in selected lesions during median follow up of 3.7 years. Importantly, clinical outcomes appeared similar between treatment strategies in the true ostial group. Severe calcification and creatinine were identified as predictors of TLF. Second, QCA assessment of follow-up angiograms over varying time intervals revealed significant lumen gain after DCB angioplasty, providing novel insights into late lumen behaviour in this anatomically complex subset. In the RCA cohort (10 DCB and 21 DES), this effect is more apparent than in the LMS lesions (7 DCB and 21 DES). However, due to the retrospective design, small sample size, and limited number of events, these findings should be considered hypothesis-generating and interpreted cautiously.

Aorto-ostial lesions are generally excluded from RCTs owing to their low prevalence (2%–3%) and marked anatomical complexity (6, 33). Consequently, evidence guiding PCI strategy derived predominantly from retrospective registries and observational studies, with outcomes of aorto-ostial stenting remaining inferior compared to those of non-ostial lesions despite advances in contemporary DES technology (33–35). These adverse outcomes are partly explained by the unique histological and anatomical characteristics, including abundant elastic and muscular tissue arising from the aortic wall, circumferential ostial muscle bundles, thick adventitia, and exposure to turbulent haemodynamic forces, all of which contribute to lesion rigidity, recoil, fibrosis, and restenosis (3, 7, 36). These factors predispose this lesion subset to increased TLF rates with both BMS and DES (11, 12, 17, 37). Our primary endpoint TLF rates following DCB treatment were 14.0% compared to 17% in DES group in this complex subset. The observed numerical difference in TLF appeared to be driven by lower rates of repeat revascularisation and target lesion myocardial infarction in the DCB cohort.

Interestingly, revascularisation rates in RCA ostium have historically exceeded those with LMS ostial disease (13, 18, 33, 38, 39). RCA ostium is frequently funnel-shaped, demonstrating greater variability in take-off angle and greater burden of nodular calcium (9, 13, 40). In contrast, LMS ostial lesions are usually larger calibre vessels with greater external elastic membrane area, larger eccentricity index and often coexist with shaft or distal bifurcation disease (41). Chronic recoil, underexpansion, geographic miss, excessive aortic protrusion, and stent deformation are consistently shown as major contributors to RCA AOL failure despite contemporary DES platforms (13, 18, 20, 39, 40, 42). Although similar mechanisms may contribute to LMS stent failure, adverse outcomes are more frequently related to larger vessel calibre, malapposition, longitudinal stent deformation and the complexity of concomitant bifurcation PCI (15, 41, 43).

In a study done by Luz et al., almost all TLR (22/23) occurred in RCA following ostial stenting using BMS and early DES, a ten-fold higher TLR risk compared to LMS (HR: 10.2 (95% CI: 1.32–75.45, *p* = 0.024) (33). Patel et al. observed no TLR in the LMS ostial group (*n* = 23) and 20% in the RCA group (*n* = 80) (16) in a study comparing long-term outcomes following IVUS vs. non-IVUS guided ostial PCI. Tsunoda et al. demonstrated IVUS-derived smaller stent cross-sectional areas and less circular ostial stent expansion in RCA had significant correlation with TLR and a 21% mean reduction in the ostial stent area (16). Of note, these studies used BMS and early generation DES. With newer generation DES, the TLR rates have improved but problems with suboptimal stenting still persists (13, 18, 42).

In contrast to previous reports, TLR patterns differed according to both treatment strategy and lesion location. No TLR events were observed in DCB-treated RCA AOLs, whereas TLR occurred in 12.7% of DES-treated RCA AOLs. Conversely, in LMS lesions, TLR rates were numerically higher following DCB than DES treatment, although this difference was not statistically significant. RCA DES failures were predominantly related to proliferative ISR and occasional stent recoil, whereas LMS failures more frequently reflected lesion-specific factors including bifurcation disease and stent-related mechanical issues. Despite the recognised importance of geographic miss in ostial stent failure, suboptimal device landing (under-landed or protruding struts) was not a significant predictor of TLF on univariate analysis in our whole cohort across LMS and RCA lesions. The study is under-powered to yield distinct differences based on treatment strategy or vessel location.

An exploratory finding of our study was the observed difference in late lumen behaviour. DCB group demonstrated significant late lumen gain in DCB group (0.25 ± 0.80), whereas DES group exhibited progressive late lumen loss during follow-up. Although angiographic follow-up was limited and numbers were modest, scatter plot analysis suggested LLG was particularly evident among RCA AOLs treated with DCBs. One possible explanation is that the absence of a permanent metallic scaffold avoids several recognised mechanisms of RCA ostial stent failure, including chronic recoil, deformation, and geographic miss. However, given the small sample size, limited angiographic follow-up, and the numerically shorter interval to follow up angiography in DCB group, this remains speculative.

It is important to recognise that patients with aorto-ostial disease represent a particularly high-risk population (5). These lesions frequently occur in older individuals with extensive coronary calcification, diffuse atherosclerotic disease, renal dysfunction, and multiple comorbidities (3, 33). Furthermore, both LMS and RCA aorto-ostial lesions may jeopardise large myocardial territories, particularly in the setting of multivessel disease. Consequently, rates of TLF and mortality remain substantial irrespective of the revascularisation strategy. In our study, increasing age and creatinine emerged as an independent predictor of all-cause mortality, emphasising that long-term prognosis in this population is influenced not only by lesion-specific factors but also by the underlying burden of systemic cardiovascular disease.

In summary, outcomes following PCI for AOLs are influenced by a complex interplay of patient-related, anatomical, hemodynamic, procedural, biomechanical and device-related factors. Many of these are inherent to the lesion or patient and are therefore not readily modifiable. Given the recognised contribution of mechanical stent failure to adverse outcomes in these lesions, it is interesting to speculate that a stent-less strategy may reduce some of these failure mechanisms.

Our findings raise the possibility that TLR rates may vary according to both vessel location and treatment strategy. While DES failure in RCA appeared predominantly associated with mechanical complications of ostial stenting, DCB strategy was associated with significant late lumen gain during follow-up. Unlike DCB- treated RCA lesions, all DCB-related TLR events occurred within the LMS cohort, which may reflect greater vessel size, procedural complexity and concomitant bifurcation disease. Although the absence of TLR events in DCB-treated RCA lesions and the observed late lumen gain provide an intriguing signal, these findings should be interpreted cautiously due to the small sample size, limited number of events, and non-protocolised angiographic follow-up. Nevertheless, the present study raises the hypothesis that a stent-less strategy may mitigate some of the mechanical limitations inherent to RCA aorto-ostial stenting while preserving future coronary access. Larger prospective studies are required to determine whether these observations represent a true treatment effect.

### 5.1 Limitations

This was a retrospective, single-centre observational study and is therefore subject to inherent selection bias and unmeasured confounding. Device strategy was not randomised and was determined by the operator at the time of the procedure; although we adjusted for clinical and angiographic characteristics, residual confounding cannot be excluded. While all consecutive cases over the study period were included to minimise referral bias, DCB experience increased gradually, which may have influenced device selection. Only Paclitaxel-coated DCB (B. Braun) was used, so the findings are not generalisable to other DCB platforms. Sample size was small, particularly within subgroup and QCA analyses, resulting in a limited number of clinical events and wide confidence intervals around effect estimates. Angiographic follow-up was opportunistic rather than protocol-mandated, with variable timing between index PCI and repeat angiography. Although QCA analysis was performed independently by two investigators experienced in QCA and demonstrated excellent inter-observer reproducibility, it was not conducted by an independent core laboratory. Furthermore, accurate angiographic assessment of aorto-ostial lesions remains inherently challenging owing to vessel overlap, ostial foreshortening, and difficulties defining reference segments. In the majority of cases, reference vessel diameter was derived from a single distal reference segment, which may have influenced quantitative measurements. Mechanistic assessment of restenosis was not systematically performed with intracoronary imaging in all patients, limiting definitive conclusions regarding the underlying mechanisms of failure. The use of intravascular imaging was modest and reflects routine UK practice during the study period rather than protocol-driven lesion preparation and may limit generalisability to contemporary practice. Nevertheless, all-cause mortality and hospitalisation follow-up was obtained through national datasets with minimal loss to follow-up, and the study represents one of the largest real-world cohorts with long-term outcomes in calcified *de novo* lesions treated with DCB or DES.

## 6 Conclusion

In this real-world cohort with aorto-ostial coronary lesions, a DCB-only strategy was feasible and associated with similar long-term outcomes to DES. Exploratory angiographic analyses demonstrated significant late lumen gain following DCB treatment. These findings are hypothesis-generating and support further prospective evaluation of a DCB approach in aorto-ostial coronary disease.

## Data Availability

The raw data supporting the conclusions of this article will be made available by the authors, without undue reservation.

## SPARTAN DCB consortium

Department of Cardiology, Norfolk and Norwich University Hospital: Timothy Gilbert, Paul Richardson, Alisdair Ryding, Trevor Wistow, Sulfi Sreekumar, Chris Sawh, Clint Maart. Department of Medicine, University of East Anglia: Johannes Reinholda, Alisdair Ryding.
